# Incidence and seasonality of respiratory viruses among medically attended children with acute respiratory infections in an Ecuador birth cohort, 2011–2014

**DOI:** 10.1111/irv.12887

**Published:** 2021-08-25

**Authors:** Eduardo Azziz‐Baumgartner, Alfredo Bruno, Michael Daugherty, Martha E. Chico, Andrea Lopez, Carmen Sofia Arriola, Domenica de Mora, Alba María Ropero, William W. Davis, Meredith McMorrow, Philip J. Cooper

**Affiliations:** ^1^ International Epidemiology and Research Team, Influenza Division U.S. Centers for Disease Control and Prevention Atlanta GA USA; ^2^ Faculty of Veterinary Medicine and Zootechnics Universidad Agraria del Ecuador Guayaquil Ecuador; ^3^ National Reference Laboratory for Influenza and Other Respiratory Viruses Instituto Nacional de Investigación en Salud Pública (INSPI) Guayaquil Ecuador; ^4^ Fundación Ecuatoriana Para Investigación en Salud Quinindé Ecuador; ^5^ School of Medicine Universidad Internacional del Ecuador Quito Ecuador; ^6^ Immunizations Program Pan American Health Organization Washington, DC USA; ^7^ Enhanced Surveillance Platforms Team Division of Viral Diseases, U.S. Centers for Disease Control and Prevention Atlanta, GA USA; ^8^ Institute of Infection and Immunity St George's University of London London UK

**Keywords:** burden, children, Ecuador, influenza, respiratory syncytial virus

## Abstract

**Background:**

Ecuador annually has handwashing and respiratory hygiene campaigns and seasonal influenza vaccination to prevent respiratory virus illnesses but has yet to quantify disease burden and determine epidemic timing.

**Methods:**

To identify respiratory virus burden and assess months with epidemic activity, we followed a birth cohort in northwest Ecuador during 2011–2014. Mothers brought children to the study clinic for routine checkups at ages 1, 2, 3, 5, and 8 years or if children experienced any acute respiratory illness symptoms (e.g., cough, fever, or difficulty breathing); clinical care was provided free of charge. Those with medically attended acute respiratory infections (MAARIs) were tested for common respiratory viruses via real‐time reverse‐transcription polymerase chain reaction (rRT‐PCR).

**Results:**

In 2011, 2376 children aged 1–4 years (median 35 months) were enrolled in the respiratory cohort and monitored for 7017.5 child‐years (cy). The incidence of respiratory syncytial virus (RSV) was 23.9 (95% CI 17.3–30.5), influenza 10.6 (2.4–18.8), adenoviruses 6.7 (4.6–28.0), parainfluenzas 5.0 (2.3–10.5), and rhinoviruses, bocaviruses, human metapneumoviruses, seasonal coronaviruses, and enteroviruses <3/100 cy among children aged 12–23 months and declined with age. Most (75%) influenza detections occurred April–September.

**Conclusion:**

Cohort children frequently had MAARIs, and while the incidence decreased rapidly among older children, more than one in five children aged 12–23 months tested positive for RSV, and one in 10 tested positive for influenza. Our findings suggest this substantial burden of influenza occurred more commonly during the winter Southern Hemisphere influenza season.

## 1 BACKGROUND

There is insufficient information about the burden and timing of respiratory virus epidemics among young children in tropical low‐ and middle‐income countries (LMICs) to adequately guide mitigation.^1^, ^2^, ^3^ Acute respiratory illnesses, typically caused by respiratory viruses, are common causes of morbidity among young children and are frequently associated with pneumonia, hospitalization, and death. Investment in their prevention and control depends, in part, on understanding their incidence and seasonality. Much is known about respiratory viruses in temperate high‐income countries; comparatively little is known from tropical LMICs where most of the mortality occurs.

For example, in 2018, influenza caused >10 million acute respiratory infections among children aged <5 years.^1^ Such annual epidemics disproportionately affect tropical LMICs. Epidemics in more northern latitudes typically occur October–March; those in southern latitudes occur April–September, but it is often unclear when epidemics occur in tropical latitudes.^4^ Understanding when epidemics occur helps inform disease burden modeling, vaccine formulation selection, and vaccination timing. In the tropics, however, influenza epidemics can seem unpredictable and their associated disease burden poorly characterized.

Like influenza, respiratory syncytial virus (RSV) causes substantial morbidity and mortality. For example, in 2015, RSV caused >30 million acute respiratory infections and an estimated 59600 in‐hospital deaths in children aged <5 years.^4^ While there are currently no RSV vaccines in use to mitigate this burden, there are several in development. Countries can use monoclonal antibodies during RSV epidemics to prevent RSV in high‐risk children, but these are typically costly and infrequently used in LMICs. Like influenza, RSV epidemics in temperate countries typically occur during cool weather; RSV seasonality in humid, tropical climates, however, is not well characterized.

Ecuador, with three geoclimatic zones (coastal, Andean, and Amazonian), has complex respiratory virus epidemics^5^ and limited burden information to justify sustained investments in mitigation.^6^ In 2006, Ecuador's Ministry of Public Health introduced influenza vaccination among pregnant women and infants aged 6 months through 5 years^7^ and subsequently recommended the use of antivirals for the treatment of persons with severe or progressive community acquired pneumonia during influenza epidemics.^8^ Ecuador is the only South American country, however, to use a Northern rather than Southern Hemisphere vaccine formulation against influenza illness^9^ in part because of uncertainty about its influenza seasonality. While Ecuador does not currently use monoclonal antibodies within the public health system to prevent severe RSV illness, it does launch annual handwashing and respiratory hygiene campaigns to prevent contagion with respiratory viruses.

To substantiate the need for investments in respiratory virus prevention and optimize the timing of interventions, we used systematically collected birth cohort data from Ecuador to (i) estimate the incidence of medically attended influenza, RSV, and other respiratory viruses; (ii) assess whether influenza incidence was highest during the April–September Southern Hemisphere season versus the October–March Northern Hemisphere season; and (iii) explore peak epidemic weeks of influenza and RSV activity.

## 2 METHODS

A description of the Ecuador Life (ECUAVIDA) study has been previously published.^10^ In brief, a prospective birth cohort was established in Quinindé, Ecuador. From 2005 to 2009, staff enrolled newborns delivered at Hospital Padre Alberto Buffoni (HPAB). Inclusion criteria were (1) healthy newborns aged <14 days; (2) stool samples collected from mothers during pregnancy for helminth infections; (3) maternal age ≥17 years; (4) anticipated residence in Quinindé for ≥2 years; and (5) accessibility of households to the study teams. Of 4087 mothers–infant pairs evaluated for eligibility, 1683 (41%) did not fulfill all eligibility criteria (e.g., 525 [31.2%] were excluded because households could not routinely access the study clinic).

On January 1, 2011, children still participating in ECUAVIDA were consented into the respiratory cohort and followed prospectively to identify medically attended acute respiratory infections (MAARIs) until their eighth birthday or the end of sampling on June 30, 2014. During the study, mothers brought children to the HPAB clinic for routine checkups at ages 1, 2, 3, 5, and 8 years or if children experienced any acute respiratory illness symptoms (e.g., cough, fever, or difficulty breathing); clinical care was provided free of charge.

Study clinicians defined MAARI as nasopharyngitis, acute otitis media, sinusitis, laryngitis, epiglottitis, tracheitis, bronchitis, bronchiolitis, or pneumonia diagnosed at HPAB. Staff collected nasopharyngeal specimens using Dacron swabs from children with MAARI at the clinic. Specimens were stored at −80°C and shipped on dry ice to Ecuador's National Reference Laboratory (NRL) for real‐time reverse‐transcription polymerase chain reaction (rRT‐PCR) detection of influenza and immunofluorescence detection of other respiratory viruses using World Health Organization (WHO) protocols.

We calculated the incidence of MAARI and MAARI‐associated respiratory viruses using previously described methods.^11^ We accounted for under‐ascertainment of influenza, RSV, adenovirus, and parainfluenza virus 1–3 among children with MAARIs who were not swabbed by multiplying their frequency by the proportion of samples testing positive for these viruses at the NRL surveillance in the same age group and epidemic week. We calculated participating children's person time as the number of days from study enrollment (January 1, 2011) to study conclusion (June 30, 2014), last completed checkup visit, or eighth birthday and excluded days when parents missed routine checkups and/or 14 days after a MAARI event. We also restricted the risk period to weeks when specific viruses were circulating in Ecuador according to NRL. Resulting rates incorporated the variance in the age‐adjusted proportion of samples testing positive per epidemic week and the sensitivity of the immunofluorescence assay used by NRL to test surveillance samples for non‐influenza viruses.^12^

To assess influenza and RSV epidemic timing,^9^ we calculated the average proportion of samples testing positive for these viruses each month; we did not assess the epidemic timing of other respiratory viruses because we did not anticipate sufficient detections for a meaningful analysis. The start of the epidemic was defined as the first month when the proportion of positive samples was greater than the annual mean for ≥2 months, and the end was defined as the first month when the proportion of influenza positive samples remained below the annual mean for ≥2 months.^13^ We also calculated the influenza rate ratio during April–September Southern Hemisphere season versus October–March Northern Hemisphere season.

The study protocol was approved by the Bioethics Committee of the Universidad San Francisco de Quito, Ecuador (Protocol 6‐11‐2010). Informed written consent for participation in the study was obtained from parents or legal guardians of children.

## 3 RESULTS

In 2011, 2376 children aged 1–4 years (median 35 months) were enrolled in the respiratory cohort and accrued 7017.5 child‐years (cy). Half (49%) of the children were female, and most (74%) were from mothers who self‐identified as Mestizo (i.e., of mixed Spanish and indigenous descent, Table 1). Twenty‐four percent had received at least one influenza vaccine between ages 6 and 24 months, and 17% had received two doses. The 1984 (84%) children who completed the study had similar demographics to those who did not; 221 (9%) moved from the study area, and 102 (4%) withdrew; 67 (3%) missed their last checkup, and two (0.1%) died because of unknown reasons.

**TABLE 1:** Demographic characteristics of children enrolled in the Quinindé respiratory cohort, 2011–2014, *N* = 2376

Characteristics		*N* (%)
Age at enrollment	12–23 months	393 (16.5)
	24–35 months	896 (37.7)
	36–95 months	1087 (45.7)
Sex	Male	1216 (51.2)
	Female	1160 (48.8)
Ethnicity	Afro‐Ecuadorian	607 (25.6)
	Mestizo^a^	1760 (74.1)
	Indigenous	9 (0.4)
Fathers with secondary or tertiary education	Yes	1196 (55.0)
	No	977 (45.0)
	Missing	203
Household monthly income (USD)	≤$85	131 (6.2)
	$86–$200	1228 (58.3)
	>$200	749 (35.5)
	Missing	268
Marital status of parents	Partnership	1757 (74.0)
	Married	343 (14.4)
	Other	276 (11.6)
Median household size		6.0 (4–7^b^)
Smoking in household	Yes	603 (25.4)
Family history of comorbidities^c^	Yes	802 (33.8)
History of pentavalent vaccine^d^ receipt at 24 months of age	Three doses	1907 (88.4)
	Two doses	140 (6.5)
	None	111 (5.1)
	Missing	218
History of any influenza vaccine receipt at 24 months of age	Two doses	322 (17.0)
	One dose	464 (24.4)
	None	1113 (58.6)
	Missing	477

^a^ Mixed Amerindian and Caucasian.

^b^ Interquartile range (IQR).

^c^ Reported history of ischemic heart disease, diabetes, high blood pressure, chronic wheezing or difficulty breathing, asthma, allergic rhinitis, or eczema among parents or siblings.

^d^ DTP‐HepB‐Hib vaccine.

Forty‐one percent (964) of children were brought to the clinic with at least one MAARI (total of 2192 MAARIs) of which 718 (74%) were successfully swabbed; 437 (45%) had one; 232 (24%) two; 121 (13%) three; 74 (8%) four; and 100 (10%) ≥5 MAARIs. Children with MAARIs were more likely to be Afro‐Ecuadorian than those who without MAARIs (relative risk 1.2, 95% CI 1.1–1.3). The most common parentally reported MAARI symptoms were cough (92%), congestion (70%), rhinorrhea (65%), fever (64%), poor appetite (50%), difficulty breathing (25%), wheezing (23%), irritability (17%), nausea (15%), and vomiting (13%). Upon examination, 20% of MAARIs were attributed to bronchitis, and 4% were prescribed antibiotics, commonly amoxicillin (56, 60%); none were prescribed antivirals.

**FIGURE 1:**
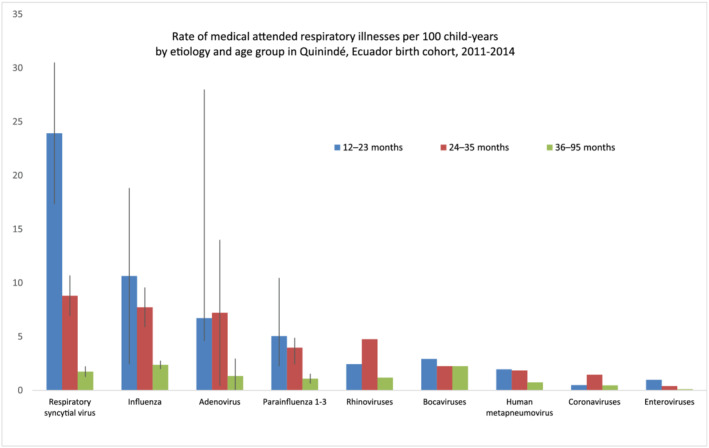
Rate of medical attended respiratory illnesses per 100 child‐years by etiology and age group in Quinindé, Ecuador birth cohort, 2011–2014. *Respiratory syncytial virus, influenza, adenovirus, and parainfluenza 1–3 rates age‐adjusted for children missing swabs, weeks when viruses were circulating throughout Ecuador, and the sensitivity of the laboratory assay. Rhinovirus, bocavirus, human metapneumovirus, coronaviruses (i.e., 1, 43, 63, and 229), and enteroviruses could not be similarly adjusted because these were not part of the National Reference Laboratory surveillance testing algorithm

Among the 964 children aged 12–95 months who were tested during a MAARI, 116 (12%) tested positive for rhinovirus, 97 (10%) influenza, 80 (8%) human bocavirus, 63 (7%) human metapneumovirus (HMPV), 61 (6%) parainfluenza 1–3, 60 (6%) RSV, 53 (5%) adenoviruses, 42 (4%) seasonal coronaviruses, and 11 (1%) enteroviruses. Eighteen percent (*n* = 18) of the 97 influenza‐positive and 27% (*n* = 16) of 60 RSV‐positive MAARIs were associated with difficulty breathing. Overall MAARI incidence was 31.1/100 cy, peaking at 95.4/100 cy at age 12–23 months and declining to 69.6/100 cy at 24–35 months, and 23.1/100cy at 36–95 months. The incidence of RSV was 23.9/100 cy (95% CI 17.3–30.5), influenza 10.6/100 cy (2.4–18.8), adenoviruses 6.7/100 cy (4.6–28.0), parainfluenzas 5.0/100 cy (2.3–10.5), and rhinoviruses, bocaviruses, HMPVs, seasonal coronaviruses, and enteroviruses <3/100 cy among children aged 12–23 months and declined with age (Figure 1) (Table 2).

**TABLE 2:** Laboratory detections, estimated illnesses among children missing swabs, person time at risk, and rates per 100 child‐years about birth cohort children in Ecuador 2011–2014

		Respiratory syncytial virus	Influenza	Adenovirus	Parainfluenza 1–3	Rhinovirus	Bocavirus	Human metapneumovirus	Coronaviruses 1, 48, 64, 226	Enterovirus
12–95 months	Laboratory detections	60	97	53	61	116	80	15	42	11
	Estimated among those missing swabs	144 (134–155)^a^	63 (54–71)	10 (−9–28)	34 (25–44)	NA	NA	NA	NA	NA
	Lab‐confirmed and estimated illnesses	204 (194–215)	101 (93–110)	63 (44–81)	95 (86–105)	NA	NA	NA	NA	NA
	Person time (years)	7044	7044	7044	7044	7044	7044	7044	7044	7044
	Proportion of person time at risk	0.80	0.77	0.43	0.81	NA	NA	NA	NA	NA
	Risk period (years)	5612	5419	3019	5728	NA	NA	NA	NA	NA
	Rate/100 child‐years	3.6 (3.5–3.8)	2.9 (2.8–3.1)	2.1 (1.5–2.7)	1.7 (1.5–1.8)	1.65	1.14	0.21	0.60	0.16
12–23 months	Laboratory detections	1	2	4	4	5	6	4	1	2
	Estimated among those missing swabs	39 (27–48)	9 (1–17)	2 (−17–20)	5 (−5–15)	NA	NA	NA	NA	NA
	Lab‐confirmed and estimated illnesses	39 (28–49)	11 (3–19)	6 (−13–24)	9 (−1–19)	NA	NA	NA	NA	NA
	Person time (years)	205	205	205	205	205	205	205	205	205
	Proportion of person time at risk	0.79	0.50	0.42	0.87	NA	NA	NA	NA	NA
	Risk period (years)	162	103	87	178	NA	NA	NA	NA	NA
	Rate/100 child‐years	23.9 (17.3–30.5)	10.6 (2.4–18.8)	6.7 (4.6–28.0)	5.0 (2.3–10.5)	2.4	2.9	1.9	0.5	1.0
24–35 months	Laboratory detections	16	25	18	20	36	17	14	11	3
	Estimated among those missing swabs	34 (23–45)	11 (2–19)	2 (−17–21)	6 (−4–16)	NA	NA	NA	NA	NA
	Lab‐confirmed and estimated illnesses	50 (39–61)	36 (7–44)	20 (1–39)	26 (16–36)	NA	NA	NA	NA	NA
	Person time (years)	756	756	756	756	756	756	756	756	756
	Proportion of person time at risk	0.72	0.61	0.37	0.87	NA	NA	NA	NA	NA
	Risk period (years)	568	462	280	654	NA	NA	NA	NA	NA
	Rate/100 child‐years	8.8 (6.9–19.7)	7.7 (5.9–9.6)	7.2 (04–14.0)	4.0 (3.1–5.5)	4.8	2.2	1.9	1.5	0.4
36–95 months	Laboratory detections	43	68	29	36	72	57	45	28	6
	Estimated among those missing swabs	41 (18–65)	48 (29–67)	5 (−36–47)	18 (−5–41)	NA	NA	NA	NA	NA
	Lab‐confirmed and estimated illnesses	84 (61–108)	116 (97–135)	341 (−7–76)	54 (31–77)	NA	NA	NA	NA	NA
	Person time (years)	6104	6104	6104	6104	6104	6104	6104	6104	6104
	Proportion of person time at risk	0.79	0.80	0.42	0.82	NA	NA	NA	NA	NA
	Risk period (years)	4836	4886	2569	4983	NA	NA	NA	NA	NA
	Rate/100 child‐years	1.79 (1.3–2.2)	2.4 (2.0–2.8)	1.3 (−0.3–3.0)	1.1 (0.6–1.5)	1.2	0.9	0.7	0.5	0.1

^a^ Values represent 95% confidence interval of the variance in the age‐adjusted proportion of surveillance samples positive for each respiratory virus on any given week and the sensitivity of the assay.

**FIGURE 2:**
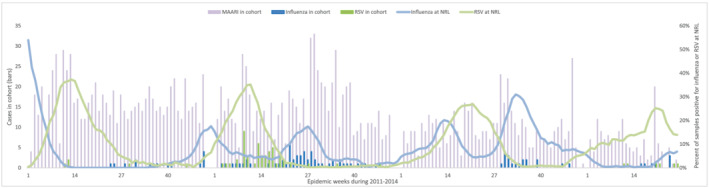
Children with laboratory‐confirmed influenza and respiratory syncytial virus (RSV) medically attended acute respiratory infection (MAARI) in the Esmeraldas Province birth cohort and percent of samples testing positive for each virus at the National Reference Laboratory (NRL) throughout Ecuador, 2011–2014

**FIGURE 3:**
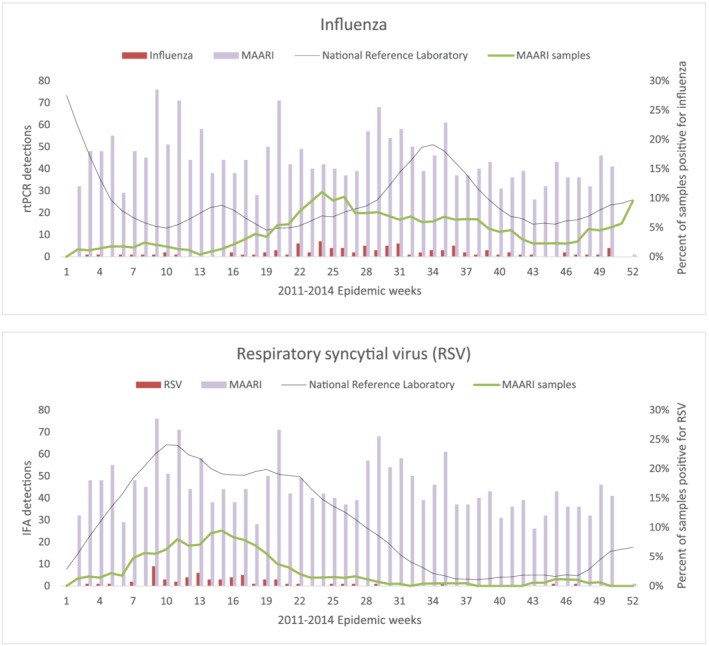
Children with laboratory‐confirmed influenza and respiratory syncytial virus (RSV) medically attended acute respiratory infection (MAARI) in the Esmeraldas Province birth cohort and weighted average percent of samples testing positive for each virus at the National Reference Laboratory (NRL) throughout Ecuador by epidemic week, 2011–2014

Children presented with MAARI year‐round, but influenza epidemics in the cohort occurred during August–October and December in 2011, May–July in 2012, July–September in 2013, and April until study conclusion in June 2014 (Figure 2). On average, influenza epidemics occurred late April through mid‐September, when 75% influenza detections occurred, and again during a secondary peak November–December, when the rest of detections occurred (Figure 3A). The incidence of influenza during April–September was 2.8 times higher than the incidence during October–March (*P* < .0001). RSV epidemics typically preceded influenza epidemics and occurred during the January–April cooler weather months in Esmeraldas Province when 82% of laboratory‐confirmed RSV were identified (Figure 3B).

## 4 DISCUSSION

Cohort children frequently had MAARIs attributable to respiratory viruses. Indeed, almost all children aged 12–23 months had MAARIs, and more than one in five tested positive for RSV and one in 10 for influenza. The incidence of respiratory viruses among this young age group is important because younger children are also at highest risk of viral^14^ and bacterial pneumonias^15^ and subsequent hospitalizations.^16^ Such a burden suggests the value of continued investment in Ecuador's pharmaceutical and nonpharmaceutical interventions to prevent respiratory illnesses, which were associated with an annual productivity loss of US$152 million.^17^

Rates of RSV among cohort children were on the higher range of those estimated for high‐income countries^18^ and similar to those estimated from the few other tropical LMICs that have estimated such rates through cohort studies (e.g., Kenya^19^). While there are currently no licensed vaccines to prevent RSV among infants, several are in development^20^ and might eventually be of value in mitigating the global burden of RSV. Monoclonal antibodies are currently available for the prevention of RSV illness among premature infants, but each dose is costly,^21^ and monoclonal antibodies are not currently included in Ecuador's list of subsidized essential drugs.^8^ Additional studies might be beneficial to explore the cost–benefit of such monoclonal antibodies for high‐risk infants in LMICs. Should providers in Ecuador choose to use monoclonal antibodies, these might be most cost‐effective if used during the peak of the January–April RSV season.

The incidence of influenza among cohort children was similar to that of other upper‐middle income countries^22^ that invest in vaccination of young children.^23^ Despite a substantial burden, annual Ministry of Public Health vaccination campaigns, and free‐of‐charge vaccination initiated in 2006,^7^ only one in six cohort children was fully vaccinated against influenza during 2011–2014. Similarly, in a 2015–2016 study in Quito, only 36.6% of pregnant women targeted for vaccination had been vaccinated against influenza; the most common reason cited for not being vaccination was lack of a recommendation from a health care provider. It is unclear, however, if current vaccinate coverage remains low in Ecuador. A recent PAHO report suggests that national vaccination coverage among Ecuador children, estimated through administrative methods, might have been >60% in 2014.^23^ Ecuador might therefore benefit from reevaluation of its vaccine program using standardized WHO tools to estimate current coverage, identify barriers to full influenza vaccination necessary for effective protection against hospitalization,^24^ and determine which steps are necessary to meet the WHO Immunization Agenda 2030 strategic goals.

In our study, 3/4 of laboratory‐confirmed influenza occurred during April–September, the typical Southern Hemisphere season. Though influenza illnesses among children in Quinindé clustered in a Southern Hemisphere pattern, one of the four study years (2011) also had an epidemic during December in a Northern Hemisphere pattern. The Quinindé findings are similar to those of a multiyear cohort in neighboring Túmbes, Peru, where influenza activity occurred during the Northern and Southern hemisphere seasons; Cuzco, Lima, and Puerto Maldonado further to the south more often had influenza activity during the April–September Southern hemisphere season.^22^ If replicable in other regions within Ecuador, such findings suggest the potential value of primarily using Southern Hemisphere vaccine formulations and schedules in Ecuador and aligns with the 2015 PAHO Technical Advisory Group recommendations to vaccinate against influenza using the latest available formulations prior to the peak of influenza illnesses. The findings also suggest the potential value of empiric treatment with antivirals during peak influenza activity that, on average, occurred in June.^25^

Our study, done in 2011–2014, found that children in Quinindé sporadically had MAARI associated with seasonal coronaviruses 1, 48, 64, and 226. Seasonal coronaviruses, while detectable, seemed to infrequently trigger health‐seeking at the study clinic especially when compared with RSV, influenza, and other commonly tested respiratory viruses. It is unclear if SARS‐CoV‐2, which has caused substantial illness in Ecuador and globally, might in the future be overshadowed by RSV. In the meantime, the Ecuador NRL continues to test for SARS‐CoV‐2, tracking the spread of COVID‐19 through its population.^26^ There may be important lessons about the use of nonpharmaceutical interventions to prevent COVID‐19 in LMICs that might have wider application for the prevention of future seasonal respiratory virus epidemics.^27^

Our study had strengths and limitations. Unlike other seasonality studies that used administrative data and modeling to estimate influenza epidemic periods,^9^ we used robust birth cohort data to calculate incidence of influenza‐associated MAARIs and assess influenza epidemic periods. Though these data were robust, our study only incorporated information from households accessible to the study clinic, during 3.5 years, and from one rural coastal district (<1% of Ecuador's population); our findings might therefore not represent influenza and RSV activity in other geoclimatic regions of the country. Last, we could not adjust the incidence of rhinovirus, bocavirus, HMPV, coronaviruses, and enterovirus, by the number of unsampled cohort children who might have tested positive for these viruses, because the NRL did not routinely test surveillance samples for these viruses.

## 5 CONCLUSION

Children in Quinindé frequently had RSV, influenza, and other respiratory virus‐associated MAARI. On average, RSV activity occurred during January–April cooler weather months and influenza during April–September, the typical months of the Southern Hemisphere influenza season. If replicable in other microclimates in Ecuador, such findings suggest the potential value of influenza vaccine formulations to prevent illness. Influenza vaccination coverage during the study period, however, seemed lower than in other Latin American countries and suggests the benefit of reassessing the vaccination program and coverage using WHO post‐introduction evaluation tools.^28^ It will also be important to assess the impact of respiratory mitigation through nonpharmaceutical interventions during the COVID‐19 pandemic, which might yield important lessons about how to better prevent seasonal respiratory virus infections in LMICs.

## CDC DISCLAIMER

The findings and conclusions in this report are those of the authors and do not necessarily represent the views of the U.S. Centers for Disease Control and Prevention. The views expressed in this article are those of the authors and do not necessarily reflect the official policy or position of the United States Government.

## INSPI DISCLAIMER

The findings and conclusions in this report are those of the authors and do not necessarily represent the views of the National Institute of Public Health Research (INSPI). The views expressed in this article are those of the authors and do not necessarily reflect the official policy or position of the Ecuadorian Government.

## AUTHOR CONTRIBUTIONS

**Eduardo Azziz‐Baumgartner:** Conceptualization; formal analysis; methodology; resources; supervision. **Alfredo Bruno:** Data curation; formal analysis; resources; supervision. **Michael Daughterty:** Data curation; formal analysis. **Martha E. Chico:** Data curation; investigation; project administration; validation. **Andrea Lopez:** Investigation; validation. **Carmen Sofia Arriola:** Conceptualization; formal analysis; methodology; supervision; validation. **Domenica de Mora:** Investigation; validation. **Alba MarÍa Ropero:** Supervision; validation. **William W. Davis:** Formal analysis; methodology; supervision; validation; visualization. **Meredith McMorrow:** Conceptualization; supervision; validation.

### PEER REVIEW

The peer review history for this article is available at https://publons.com/publon/10.1111/irv.12887.

## Data Availability

Data available on request from the authors.
